# Mutational landscape of nasopharyngeal carcinoma based on targeted next-generation sequencing: implications for predicting clinical outcomes

**DOI:** 10.1186/s10020-022-00479-4

**Published:** 2022-05-13

**Authors:** Zihan Zhou, Peifeng Li, Xianbin Zhang, Juan Xu, Jin Xu, Shui Yu, Dongqing Wang, Wei Dong, Xiujuan Cao, Hongjiang Yan, Mingping Sun, Xiuping Ding, Jun Xing, Peng Zhang, Limin Zhai, Tingyong Fan, Shiyu Tian, Xinhua Yang, Man Hu

**Affiliations:** 1 Department of Oncology, Weifang Medical University, Weifang, Shandong China; 2 Department of Radiation Oncology and Shandong Provincial Key Laboratory of Radiation Oncology, Shandong Cancer Hospital and Institute, Shandong First Medical University and Shandong Academy of Medical Sciences, Jinan, Shandong China; 3 Department of Pathology, The 960Th Hospital of PLA, Jinan, China

**Keywords:** Gene, Tumor mutational burden, Risk score, Nasopharyngeal carcinoma, Distant metastasis-free survival, Target next-generation sequencing

## Abstract

**Background:**

The aim of this study was to draw a comprehensive mutational landscape of nasopharyngeal carcinoma (NPC) tumors and identify the prognostic factors for distant metastasis-free survival (DMFS).

**Methods:**

A total of forty primary nonkeratinizing NPC patients underwent targeted next-generation sequencing of 450 cancer-relevant genes. Analysis of these sequencing and clinical data was performed comprehensively. Univariate Cox regression analysis and multivariate Lasso-Cox regression analyses were performed to identify factors that predict distant metastasis and construct a risk score model, and seventy percent of patients were randomly selected from among the samples as a validation cohort. A receiver operating characteristic (ROC) curve and Harrell’s concordance index (C-index) were used to investigate whether the risk score was superior to the TNM stage in predicting the survival of patients. The survival of patients was determined by Kaplan–Meier curves and log-rank tests.

**Results:**

The twenty most frequently mutated genes were identified, such as *KMT2D*, *CYLD*, and *TP53* et al. Their mutation frequencies of them were compared with those of the COSMIC database and cBioPortal database. N stage, tumor mutational burden (TMB), *PIK3CA*, and *SF3B1* were identified as predictors to build the risk score model. The risk score model showed a higher AUC and C-index than the TNM stage model, regardless of the training cohort or validation cohort. Moreover, this study found that patients with tumors harboring PI3K/AKT or RAS pathway mutations have worse DMFS than their wild-type counterparts.

**Conclusions:**

In this study, we drew a mutational landscape of NPC tumors and established a novel four predictor-based prognostic model, which had much better predictive capacity than TNM stage.

**Supplementary Information:**

The online version contains supplementary material available at 10.1186/s10020-022-00479-4.

## Background

Nasopharyngeal carcinoma (NPC) is relatively uncommon. There were approximately 129,000 new patients worldwide in 2018, accounting for only 0.7% of all cancers diagnosed. The geographical global distribution of NPC is extremely unbalanced; > 70% of new cases are in eastern and southeastern Asia (Chen et al. 2019). Even though local and regional controls have been substantially improved in NPC with extensive use of combined chemotherapy in the contemporary era of intensity-modulated radiotherapy, distant metastasis has become the major cause of treatment failure and cancer-related death (Pan et al. 2016).

Currently, the tumor‐lymph node‐metastasis (TNM) staging system is the key clinical tool for prognostication, risk stratification, and making treatment decisions. However, the TNM stage is unable to accurately predict whether patients will have distant metastasis. Over the past decades, with the continuous development of sequencing technology, great efforts have been made to search for molecular biomarkers associated with distant metastasis, such as long noncoding RNA, EBV DNA, microRNA and gene expression (Chen et al. 2019; Liu et al. 2012; Tang et al. 2018; Wen et al. 2018). Efforts to promote molecular classifications are more comprehensive and becoming more prevalent in clinical cancer management (Lim and Ma 2019).

Next-generation sequencing (NGS), also called massive parallel sequencing, was developed in the last decade. It can be used not only in research settings but also in clinical practice; however, whole exome sequencing (WES) or whole genome sequencing (WGS) provide more information than what can be practically used (Xuan et al. 2013). It is difficult to process this amount of data at a level required for clinical applications, and it is too costly for individual patient diagnosis. Based on this, targeted next-generation sequencing has been developed to obtain genomic data in a timely and cost-effective way by testing clinically important genes (Nagahashi et al. 2019). In recent years, targeted next-generation sequencing has been adopted in many cancers, such as lung cancer, gastric cancer and brain tumors (Cai et al. 2019; Kneuertz et al. 2020; Sahm et al. 2016). The application of targeted next-generation sequencing to replace WGS and WES technology for genetic testing in clinical practice has become a recent trend.

The aim of this study was to draw a comprehensive mutational landscape of 40 NPC tumors and identify the prognostic factors for metastasis-free survival. Moreover, the study found combined tumor mutational burden (TMB), genomic and N stage to generate a model with more accurate prediction than TNM stage for distant metastasis-free survival (DMFS).

## Methods

### Patients and samples

Between March 2014 and August 2018, a total of 40 patients diagnosed at Shandong Cancer Hospital and Institute, Shandong First Medical University and Shandong Academy of Medical Sciences were enrolled. All of the patients were Asians and had long-term residence in Shandong Province, China. A flow chart for study design was included in Additional file 2: Fig. S1. The inclusion criteria were age over 18 years, and pathologically confirmed primary nonkeratinizing NPC. The exclusion criteria were as follows: incomplete clinical follow-up data, presence of other malignant tumors and refusal to consent to study participation. The patients were strictly followed-up and reviewed regularly until March 14, 2021. Distant metastases were defined as clinical evidence of distant disease based on clinical and radiographic findings. The clinicopathological characteristics of these patients were obtained from the electronic records of the patients. This study complied with the principles set forth in the Declaration of Helsinki. It was approved by the Institutional Review Board of Shandong Cancer Hospital and Institute, Shandong First Medical University and Shandong Academy of Medical Sciences. Written informed consent was obtained from each patient.

### DNA extraction and library preparation

DNA from formalin-fixed, paraffin-embedded (FFPE) tumor samples was extracted by a DNA Extraction Kit (QIAamp DNA FFPE Tissue Kit) according to the manufacturer's protocols. A minimum concentration of 50 ng was used for each 40 mm FFPE tumor sample. All tumor samples were sent to the laboratory (Shanghai OrigiMed Co., Ltd.) for genomic DNA extraction and hybridization capture. All coding exons of 450 genes and introns of 39 genes were incorporated into the custom hybridization capture panel. The 450 genes were considered tumor-associated genes or the upstream and downstream parts of the tumor-related pathway, and the 39 genes were frequently identified in gene rearrangements. To ensure high efficiency of capture in the low-read depth region, the probe density was increased. Libraries were each diluted to 1.05 nmol/L and then sequenced with a mean coverage of 1101 × for FFPE samples on an Illumina NovaSeq Platform (Illumina Incorporated)(Cao et al. 2019).

### Bioinformatics analysis

Reads were aligned to the human genome reference sequence (hg19) by Burrows–Wheeler Aligner, and PCR duplicates were removed using Picard. After quality recalibration and realignment using GATK, single-nucleotide variants (SNVs) and short indels were identified by MUTECT. Next, short indels were calibrated by the Pindel results. To normalize the read depths within the target regions, EXCATOR was used(Tong et al. 2021). We calculated the log-ratio per region of each gene and detected copy number variants (CNVs) by customized algorithms. Germline variants were identified by HaplotypeCaller from the Genome Analysis Toolkit (GATK v.3/3) in gvcf mode with default settings, and only those present in both normal and tumor samples were retained. Tumor cellularity was estimated by allele frequencies of sequenced SNPs. Notably, a customized algorithm can be used to detect gene rearrangements, fusions, and long indels. This panel has been stringently validated and is reliable for use in the clinic. Thirty-three cell lines and 208 clinical FFPE samples used performed to assess the concordant results between NGS and Sanger sequencing, IHC, FISH, and PCR(Cao et al. 2019).Tumor mutation burden was defined as the number of all somatic base substitutions and indels per mega base excluding synonymous mutations(Cao et al. 2019). At a minimum, five reads were required to support alternative calling.

### Statistical analysis

The statistical analysis and the graph were performed with R software (version 4.0.3), SPSS 23.0 (IBM SPSS Statistics) and GraphPad Prism 8.0 (GraphPad Software, Inc., San Diego, CA). Venn diagrams (http://bioinformatics.psb.ugent.be/webtools/Venn/) of the high frequency mutations genes were drawn for the different databases. X-title plots were used to generate an optimal cutoff value of TMB. To identify metastasis-related factors, univariate Cox regression analysis was performed. The criterion of *P* < 0.02 was selected as the filtering threshold. Next, Lasso-Cox regression analysis was used to filter the prognostic genes and construct a risk score model. Seventy percent of patients were randomly selected from the samples as a validation cohort using the R package “caret”. ROC curves and Harrell’s concordance index (C-index) were used to investigate whether the risk score was superior to the TNM stage in predicting the survival of patients. The survival of patients was determined by Kaplan–Meier curves and log-rank tests. *P*-value of less than 0.05 were considered significant.

## Results

### The clinicopathological characteristics of the patients

In total, forty patients were enrolled in this study, including 5 cases in stage I-II (stage I, n = 3; stage II, n = 2) and 35 cases in stage III–IV (stage III, n = 18; stage IV, n = 17).The median age was 47 years old at diagnosis (range from 18 to 71 years). With a median follow-up of 2.79 years (range 2.55–7.04 years), 10 patients developed distant metastases, 1 experienced local recurrence, and 1 was died. The clinicopathological characteristics of the patients are presented in Table 1.

**Table 1:** Patient characteristics

Characteristics	N (%)
Age	
< 50	23 (57.5)
≥ 50	17 (42.5)
Gender	
Female	9 (22.5)
Male	31 (77.5)
Smoking	
Yes	17 (42.5)
No	23 (57.5)
Drinking	
Yes	15 (37.5)
No	25 (62.5)
T stage	
T_1-2_	24 (60)
T_3-4_	16 (40)
N stage	
N_0-1_	10 (25)
N_2-3_	30 (75)
Recurrence	
Yes	1 (2.5)
No	39 (97.5)
Metastasis	
Yes	10 (25)
No	30 (75)
Death	
Yes	1 (2.5)
No	39 (97.5)
Prognostic stage	
I	3 (7.5)
II	2 (5)
III	18 (45)
IV	17 (42.5)

Data given are numbers

Percentages are given between brackets (%)

### Landscape of mutations

**Fig. 1:**
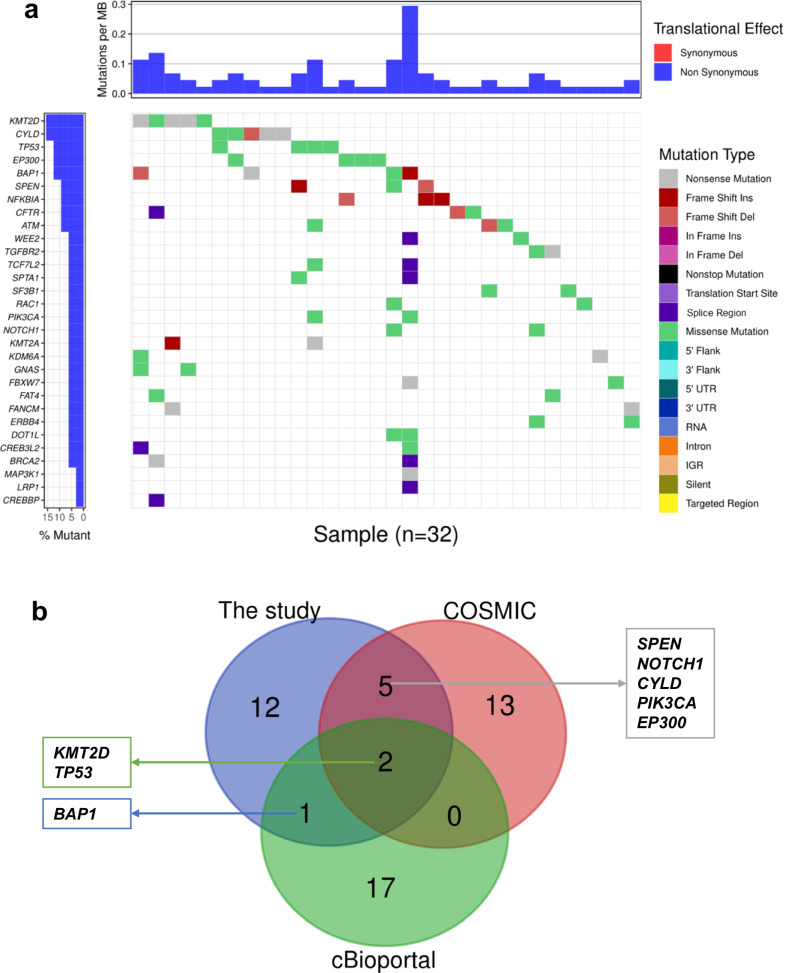
Significantly mutated genes of nasopharyngeal carcinoma (NPC). **a** Waterfall plot of the mutations among NPC patients. **b** The twenty most frequently mutated genes were compared to the COSMIC database and cBioPortal database

A total of 171 somatic mutations were detected in 123 genes, including 112 missense_mutations, 20 nonsense_mutations, 15 frame_shift_deletions, 14 splice_regions, 6 frame_shift_insertions, 3 splice_sites and 1 in_frame_ins. The median number of mutations per patient was 3 (range 0–24). Thisreveals a relatively low mutational rate and wide mutational diversity (Additional file 2: Fig. S2a, b). Missense mutations were the major mutation types and C > T transitions were the dominant aberrations, accounting for 64.29% and 71.34% of the total somatic SNVs, respectively. In addition, we noticed that the transition/transversion (Ti/Tv) ratio was 1.8 (Additional file 2: Fig. S2c-e). The top 30 genes with the most frequent mutations were found in 32 patients. Among these, the 5 most frequently mutated genes were *CYLD* (12.50%), *KMT2D* (12.50%), *TP53* (10.00%), *BAP1* (10.00%) and *EP300* (10.00%) (Fig. 1a).

The twenty most frequently mutated genes were compared to the COSMIC database (https://cancer.sanger.ac.uk/cosmic) and cBioPortal database (http://cbioportal.org). As expected, *KMT2D* and *TP53* had a high frequency of mutations in the 3 datasets. Mutations in other frequently mutated genes in our study, including *SPEN*, *NOTCH1*, *CYLD*, *PIK3CA*, and *EP300* were covered by the COSMIC database. In addition, the *BAP1* mutation frequency was high in this study and in the cBioPortal database but not high enough in the COSMIC database.

Notably, this study identified a number of significant correlations including patterns of co-occurrence or mutuality between different mutant genes especially one mutually co-occurring pair (*BAP1* and *DOT1L*) (corrected *P* < 0.05, Additional file 2: Fig. S2f-g).

### Copy number variation detection and rearrangement

**Fig. 2:**
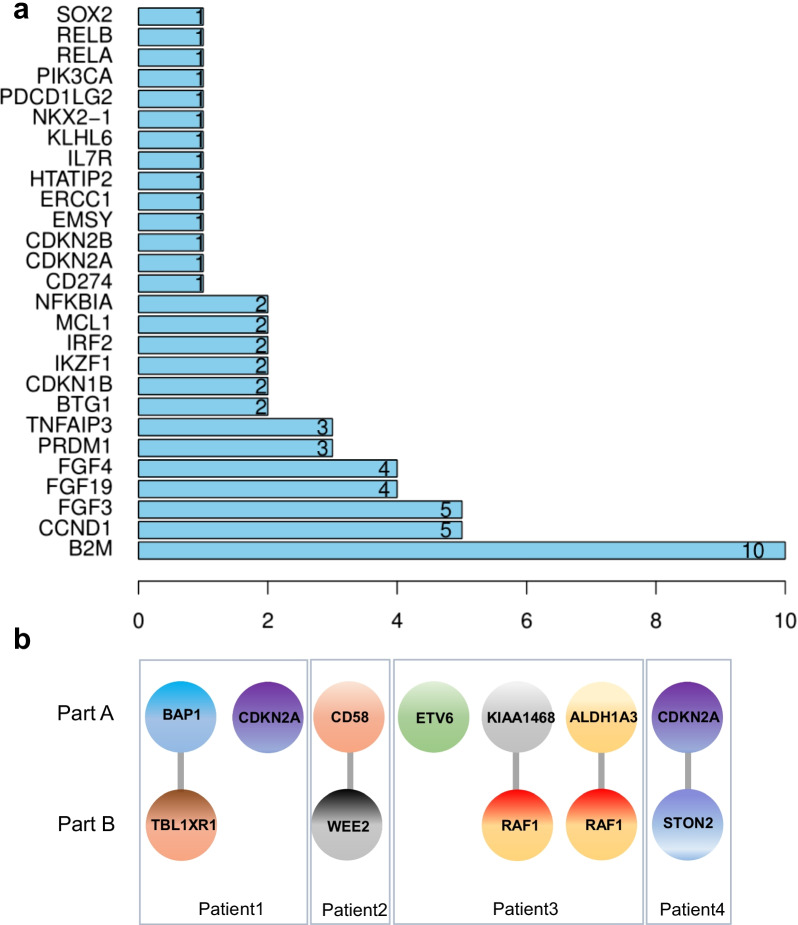
Copy number alterations and fusion/rearrangement. **a** Frequency of copy number variation per gene. **b** All of the fusion and rearrangement information

The frequency of copy number variation per gene is shown in Fig. 2a. Deletions were found in 3 genes (*NKX2-1*, *CDKN2B*, *CDKN2A*), while amplifications were detected in the other 24 genes. Among them, the deletion of *CDKN2A* and deletion of *CDKN2B* were found in the same patient, who developed bilateral pulmonic metastases. The eight CNV genes according to frequency included *B2M* (25.00%*)*, *CCND1* (12.50%), *FGF3* (12.50%), *FGF19* (10.00%), *FGF4* (10.00%), *TNFAIP3* (7.50%), and *PRDM1* (7.50%).

In addition to SNV and CNV, four patients were profiled for fusion/rearrangement of 8 genes, of which 5 genes were fusion transcripts. The study identified *CD58*-*WEE2* in Patient 2, who developed multiple bone metastases. *CDKN2A*-*STON2* was identified in Patient 4, who developed multiple bone metastases, and liver metastases. All of the fusion and arrangement information is presented in Fig. 2b.

### Enrichment of mutated genes by GO and KEGG analysis

**Fig. 3:**
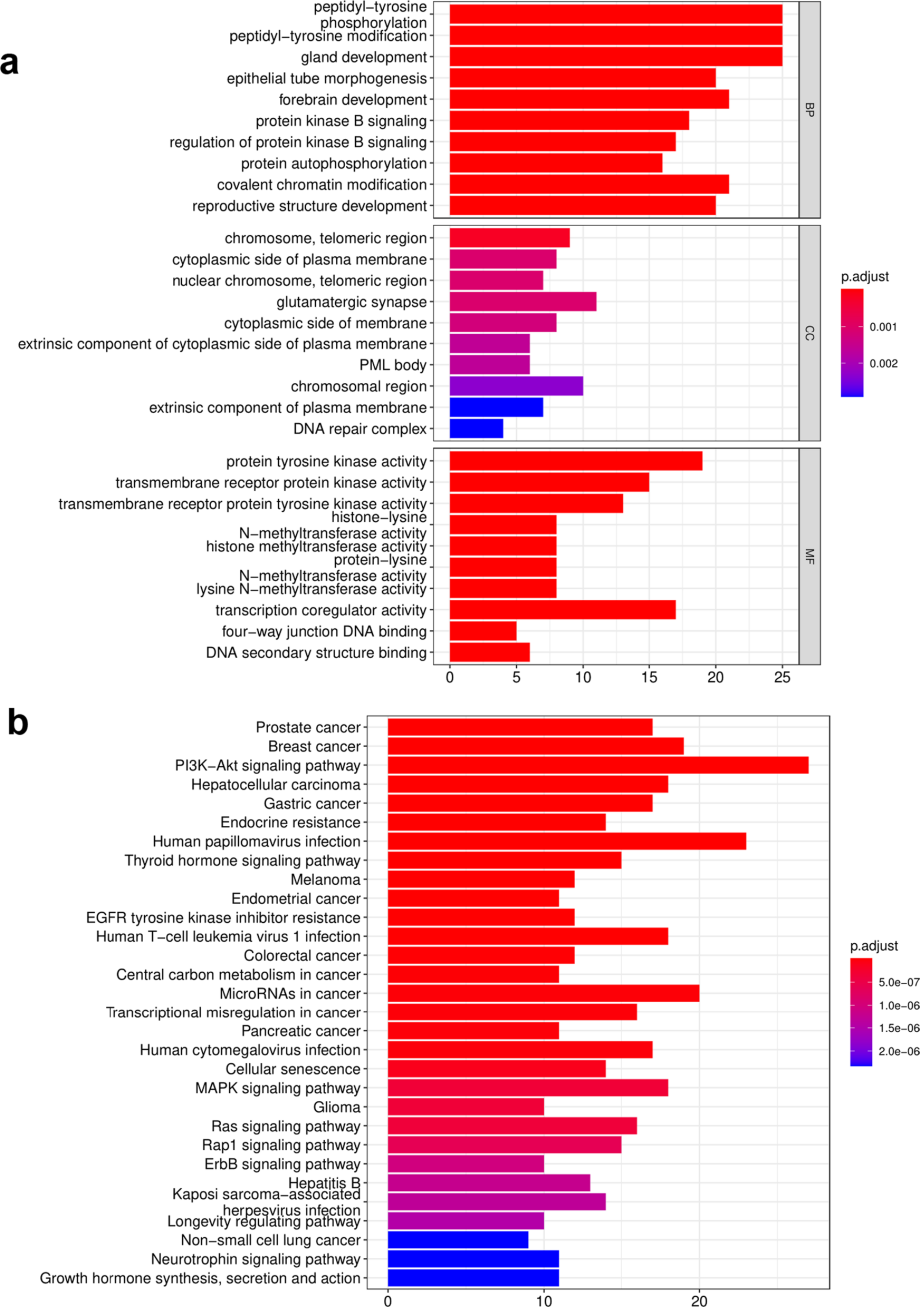
Enrichment of mutated genes by GO and KEGG analysis. **a** GO enrichment analysis. **b** KEGG enrichment analysis. Gene count: the number of mutated genes enriched in this pathway or functional term

GO and KEGG pathway analyses for all mutated genes were performed. The top 30 most significantly enriched GO and KEGG terms according to gene count and *P* value are presented in Fig. 3a, b, respectively. Regarding the biological process of GO, the genes were significantly enriched in ‘peptidyl-tyrosine phosphorylation’. Concerning the cellular component of GO, the genes were specifically focused on ‘glutamatergic synapses’. In addition, the genes were primarily assembled in ‘protein tyrosine kinase activity’ based on the molecular function of GO (Fig. 3a).

**Fig. 4:**
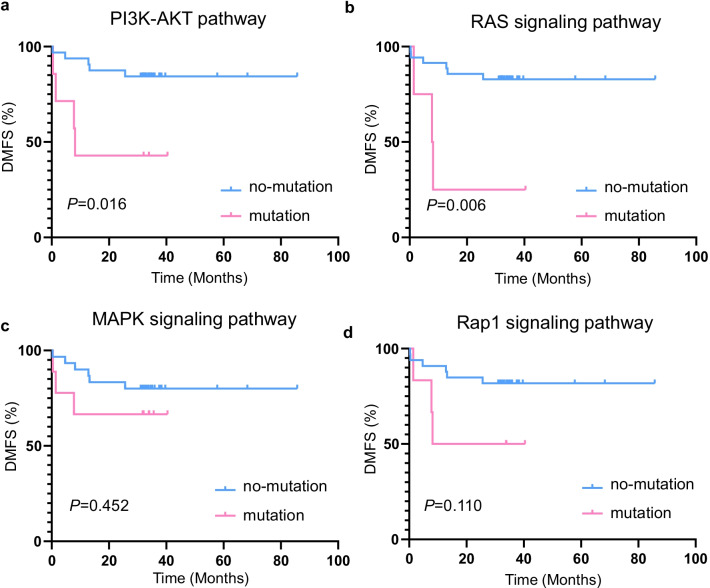
Kaplan–Meier estimates comparing distant metastasis-free survival of patients with nasopharyngeal carcinoma (NPC). **a** With and without an altered PI3K-Akt signaling pathway. **b** With and without an altered Ras signaling pathway. **c** With and without an altered MAPK signaling pathway. **d** With and without an altered Rap1 signaling pathway

Many KEGG pathways were significantly enriched, such as the PI3K-Akt signaling pathway, MAPK signaling pathway, Ras signaling pathway, Rap1 signaling pathway, cellular senescence, EGFR tyrosine kinase inhibitor resistance, and other well-known pathways. Among these, the PI3K-Akt signaling pathway was the most significantly enriched pathway, which involved in 27 genes. Next, the analysis focused on high-frequency mutations that caused amino acid changes. We found that patients with PI3K/AKT pathway or RAS pathway mutations had worse DMFS than their wild-type counterparts (*P* = 0.016, *P* = 0.006, respectively; (Fig. 4a, b)). We also the MAPK signaling pathway and Rap1 signaling pathway, they were not statistically significant (Fig. 4c, d).

### Risk score and prognostic analyses

**Fig. 5:**
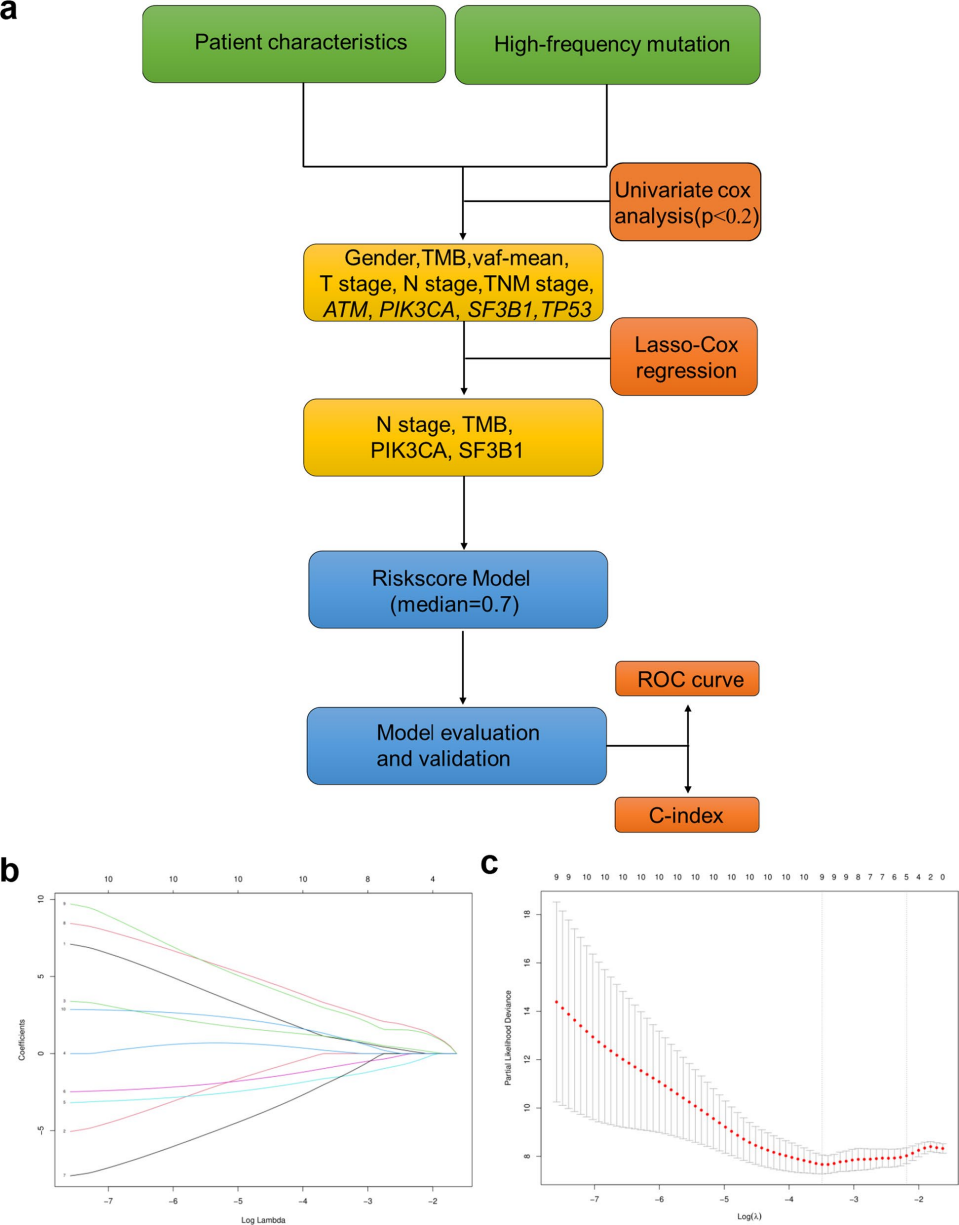

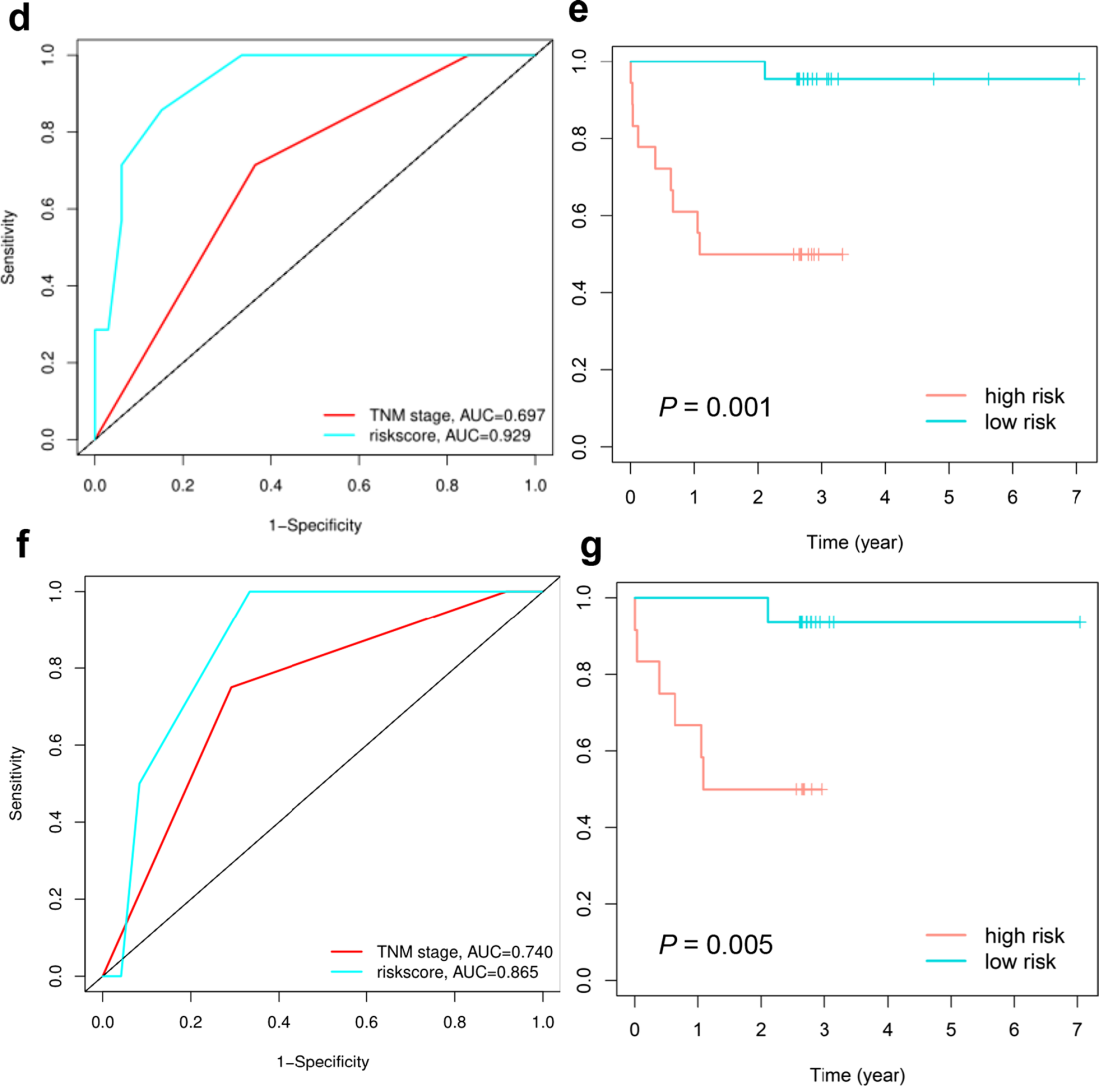
The risk score had a high predictive power for DMFS in patients with NPC. **a** Overall analysis workflow. **b** Lasso coefficient profile plots showing that the variations in the size of the coefficients of parameters shrink with an increasing value of the k penalty. **c** Penalty plot for the Lasso model with error bars denoting the standard errors. **d**, **f** ROC curves of TNM stage and risk score in the training cohort and validation cohort. **e**, **g** Kaplan–Meier analysis of the risk score model in the training cohort and validation cohort

A flowchart of the analysis workflow is illustrated in Fig. 5a. Univariate Cox regression analysis identified 9 metastasis-related factors, including sex, TMB, vaf_mean_, T stage, N stage, TNM stage, *ATM*, *PIK3CA*, *SF3B1*, and *TP53*, as potential prognostic indicators of DMFS. After primary filtering, a Lasso-Cox regression analysis was performed to further narrow down the screening results and identified TMB, N stage, *PIK3CA* and *SF3B1* as predictors for model construction (Fig. 5b, c). Risk scores for the four-predictor-based model were calculated by the ‘survival’ package. Using ROC analysis, the risk score output a higher AUC value (AUC = 0.929) than current TNM stage (AUC = 0.697; Fig. 5d). The risk score (C-index = 0.87) also showed a higher C-index than the current TNM stage (C-index = 0.70). Finally, Kaplan–Meier survival analysis showed clearly separated curves, and the DMFS of patients whose risk score was no greater than 0.7 was longer than that of patients with a risk score greater than 0.7 (*P* = 0.001; Fig. 5e).

For validation of the model, the same calculation was performed using data from our validation cohort. In the validation cohort, the risk score also displayed significantly higher AUC and C-index values than those of the TNM stage (0.865 versus 0.740, 0.833 versus 0.738; Fig. 5f). Similarly, the risk score is capable of separating the patients clearly in the validation cohort (*P* = 0.005; Fig. 5g).

### The genes related to distant metastasis in NPC

**Fig. 6:**
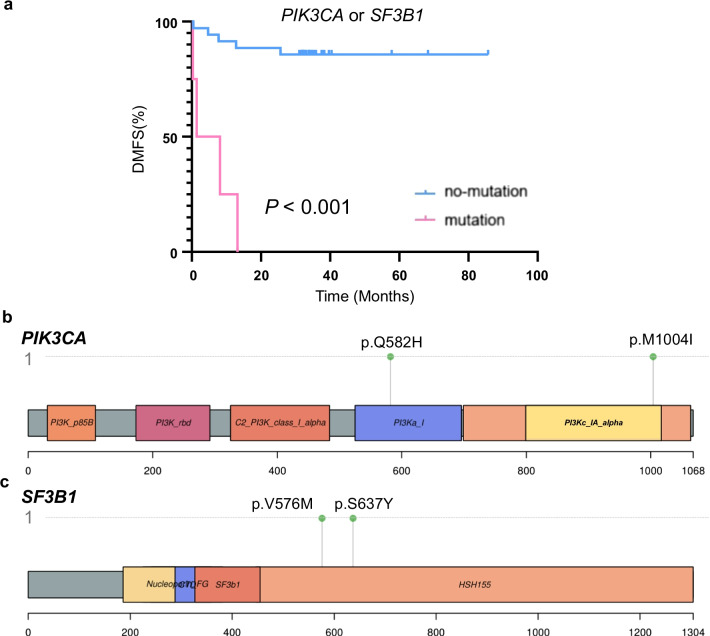
Genes associated with distant metastasis. **a** Kaplan–Meier survival curves of NPC subjects either with or without gene mutations in the *PIK3CA* or *SF3B1*. The log-rank test was used to calculate statistical significance. **b**, **c** Lollipop plot that depicts the protein mutational distribution and domains for *PIK3CA* and *SF3B1*

This study found that *PIK3CA* and *SF3B1* mutations were associated with NPC distant metastasis. Patients with *PIK3CA* and *SF3B1* mutations had shorter DMFS than patients with no-mutations (*P* < 0.001; Fig. 6a). Focusing on *PIK3CA*, one missense mutation site was located in PI3Kc_IA_alpha functional region, and glutamine was mutated to histidine here. Besides, In addition, methionine was mutated to isoleucine in the PI3Ka_I functional region (Fig. 6b). For *SF3B1*, two missense mutation sites were located in the HSH155 functional region with a dense distribution, and the amino acid variations were shown to be p.V576 M (mutation of valine to methionine) and p.S637Y(mutation of serine to tyrosine), respectively (Fig. 6c).

## Discussion

With NGS technology, the genomic landscapes of NPC have been comprehensively elucidated at the whole-exome or whole-genome levels (Tsang et al. 2020). Considering both the cost and detection rate, large targeted next-generation sequencing can be a comparable alternative to WES or WGS in most clinical cases. We found no significant differences in high-frequency mutations between our study and others. For example, the findings revealed that missense mutations, SNPs, and C > T mutations were the common mutation forms, and similar percentages were found in the COSMIC database. Unsurprisingly, *KMT2D* and *TP53* were the most frequently mutated genes in the 3 datasets. However, high-frequency mutated genes in this study and COSMIC datasets, including *SPEN*, *NOTCH1*, *CYLD* and *EP300* had no mutation frequencies in the cBioPortal database. This might be because the data in cBioPortal were relatively limited, with a sample of only 56 patients, all from the same hospital. Our analyses of CNV found that *FGF19*, *FGF4*, *FGF3* and *CCND1* gene amplification occurred in the same 4 patients. With this, we can speculate about correlations among these four genes.

To better understand the functions of these mutated genes, we conducted KEGG and GO distribution analysis. Previous studies revealed that the PI3K/AKT and MAPK signaling pathway are implicated in several biological processes, such as cellular proliferation and metastasis (Ersahin et al. 2015; Tomić et al. 2017). Our results showed that the PI3K-Akt and MAPK signaling pathways were enriched for multiple genes, and may play critical roles. Similar to findings in other studies, patients harboring at least one of the PI3K-Akt signaling pathway mutations, including *PIK3CA*, *RAC1*, and *TP53*, had worse DMFS than their wild-type counterparts (Zhang et al. 2017; Lin et al. 2014; Zheng et al. 2016). For MAPK signaling pathway mutations, the survival curves demonstrated a decreasing trend toward decreased, but the changes were modest and not statistically significant (*P* = 0.452). Moreover, we found that tumors with Ras signaling pathway mutations had worse DMFS than their wild-type. Therefore, our findings agree with Tsang et al., who that small molecule RAS inhibitors can be tried in patients with NPC (Tsang et al. 2020; O'Bryan 2019).

With recent advances in molecular biology and genome sequencing, many scholars have put immense effort into identifying molecular biomarkers to improve prognostication accuracy, such as EBV DNA, microRNA, and mRNA (Tang et al. 2018; Liu et al. 2014). Concurrently, they found that a molecular signature integrated with clinical indicators can often improve upon the prognostic capability. Molecular-based prediction models were previously constructed based on microRNA or mRNA expression, but their clinical practicability is not sufficient(Tang et al. 2018). We hoped that, with targeted NGS, we can construct a risk score model of NPC that is easier to incorporateinto clinical practice. The risk score was established based on two genes (*PIK3CA* and *SF3B1*), TMB, and N stage to identify high-risk prognostic individuals with a worse DMFS. Obviously, the risk score model had better value for predicting metastasis than does the TNM stage.

Remarkably, we found that *PIK3CA* and *SF3B1* mutations were independent prognostic factors. K-M analyses showed that patients with *PIK3CA* or *SF3B1* mutations had a better prognosis in terms of DMFS. As a master regulator of cancer, the importance of the PI3K pathway is self-evident (Yang et al. 2019). *PIK3CA*, which is a key gene in multiple solid tumors, including NPC, is known to activate the PI3K pathway (Madsen et al. 2018; Mjos et al. 2017). After the first PI3K inhibitor (alpelisib) was approved by the FDA to treat breast cancer patients with *PIK3CA*-mutations, people expected precision-based PI3K inhibitors to would be beneficial to *PIK3CA*-mutated NPC patients in the future (Tsang et al. 2020; Zhang et al. 2020). *PIK3CA*, as an independent predictor, was not surprising. Interestingly, we are the first group to identify *SF3B1* as a potential novel biomarker for predicting DMFS in NPC. Mutations in *SF3B1* have been identified at a relatively high frequency in some tumors, such as hematologic malignancies, uveal melanoma (UM), and breast cancers (BC) (Ellis et al. 2012; Banerji et al. 2012; Cazzola et al. 2013). The majority of studies reported that *SF3B1* mutations conferred a favorable prognosis; however, *SF3B1*-mutant UM was reported to be prone to metastasis (Yavuzyigitoglu et al. 2016). Moreover, *SF3B1* mutation can dysregulate the NF-κB pathway in CLL (Wang et al. 2016), which was persistently activated by somatic gene alterations or viral oncoproteins that have been shown to play a crucial role in NPC tumorigenesis (Tsang et al. 2020; Zeligs et al. 2016). Further insights regarding the functional role of *SF3B1* in NPC might offer fundamental evidence for DMFS prediction of NPC in the future.

The major limitation of the present study is the small sample size and the absence of a validation cohort. To reduce the sample error, we used univariate Cox regression analysis and multivariate Lasso-Cox analysis. In the future, the sample size will be enlarged and the follow-up duration will be extended to confirm the results of this study.

## Conclusions

In this study, we drew a mutational landscape of NPC tumors and established a novel four predictor-based prognostic model, including N stage, TMB, *PIK3CA* and *SF3B1*. The model had much better predictive capacity than TNM stage.

## Supplementary Information

**Additional file 1: Fig. S1.** Flow chart for study design.

**Additional file 2: Fig. S2.** Mutational Landscape of NPC. (a-e) Mutational patterns and proportion of genetic alterations in nasopharyngeal carcinoma. (f) Statistically significant mutual exclusivity or co-occurrences among the identified genes using pairwise Fisher’s exact test. (g) Oncostrip plot showing the detailed co-occurrences of *BAP1* and *DOT1 L* in NPC.

## Data Availability

Mutation frequency data were derived from COSMIC database (https://cancer.sanger.ac.uk/cosmic) and cBioPortal database (http://cbioportal.org), other raw data is available from the corresponding author on reasonable request.

## Abbreviations

NPC: Nasopharyngeal carcinoma
DMFS: Distant metastasis-free survival
ROC: Receiver operating characteristic
C-index: Concordance index
NGS: Next-generation sequencing
WES: Whole exome sequencing
WGS: Whole genome sequencing
TMB: Tumor mutational burden
FFPE: Formalin-fixed, paraffin-embedded
SNVs: Single-nucleotide variants
CNVs: Copy number variants
