# Supplementary figures and images for: Mutational landscape of nasopharyngeal carcinoma based on targeted next-generation sequencing: implications for predicting clinical outcomes
Source: Mol Med. 2022 May 13;28:55. doi: 10.1186/s10020-022-00479-4 (PMC9107145; doi:10.1186/s10020-022-00479-4)

## Supplemental Figure 1

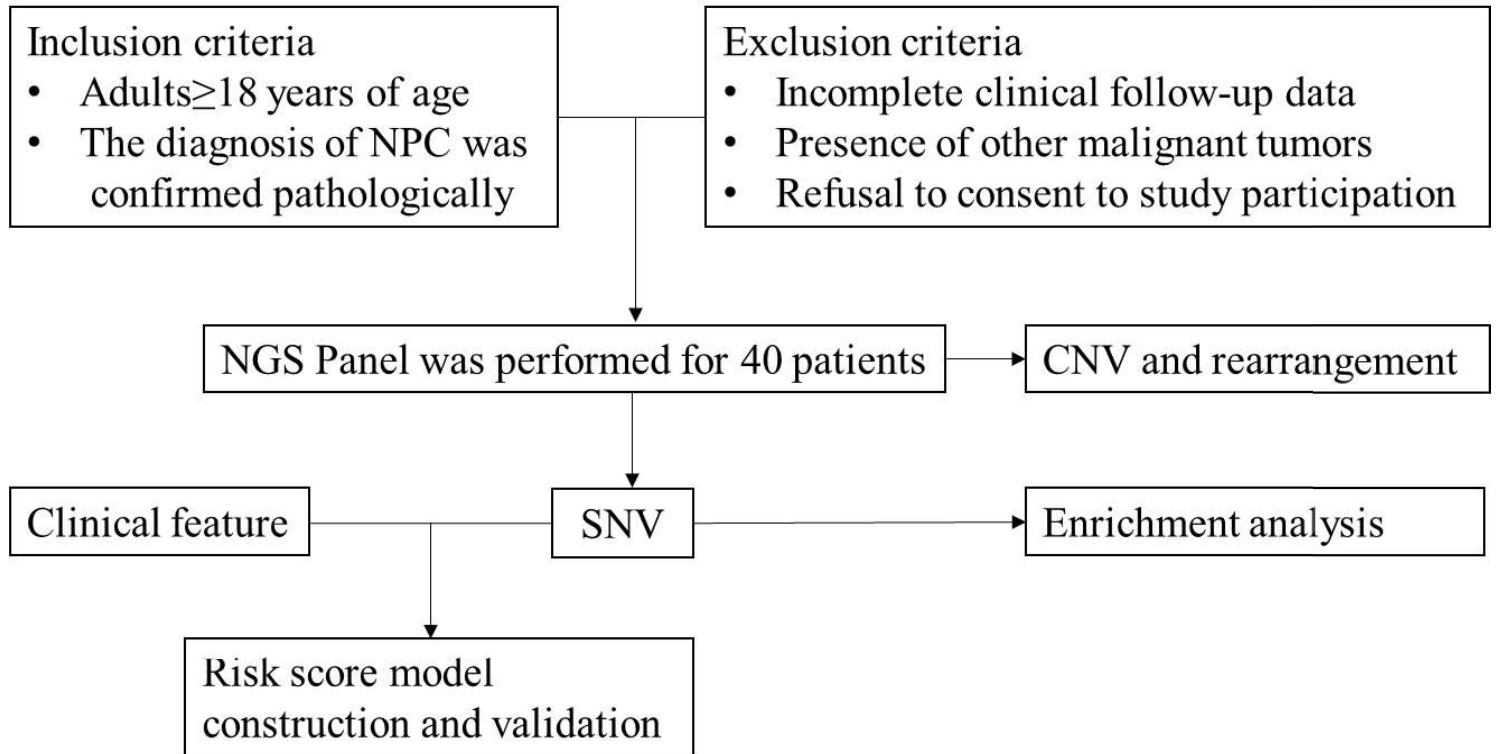

Supplement: Supplementary file 1 — Additional file 1: Fig. S1. Flow chart for study design. [file 10020_2022_479_MOESM1_ESM.pdf]

# Supplemental Figure 2

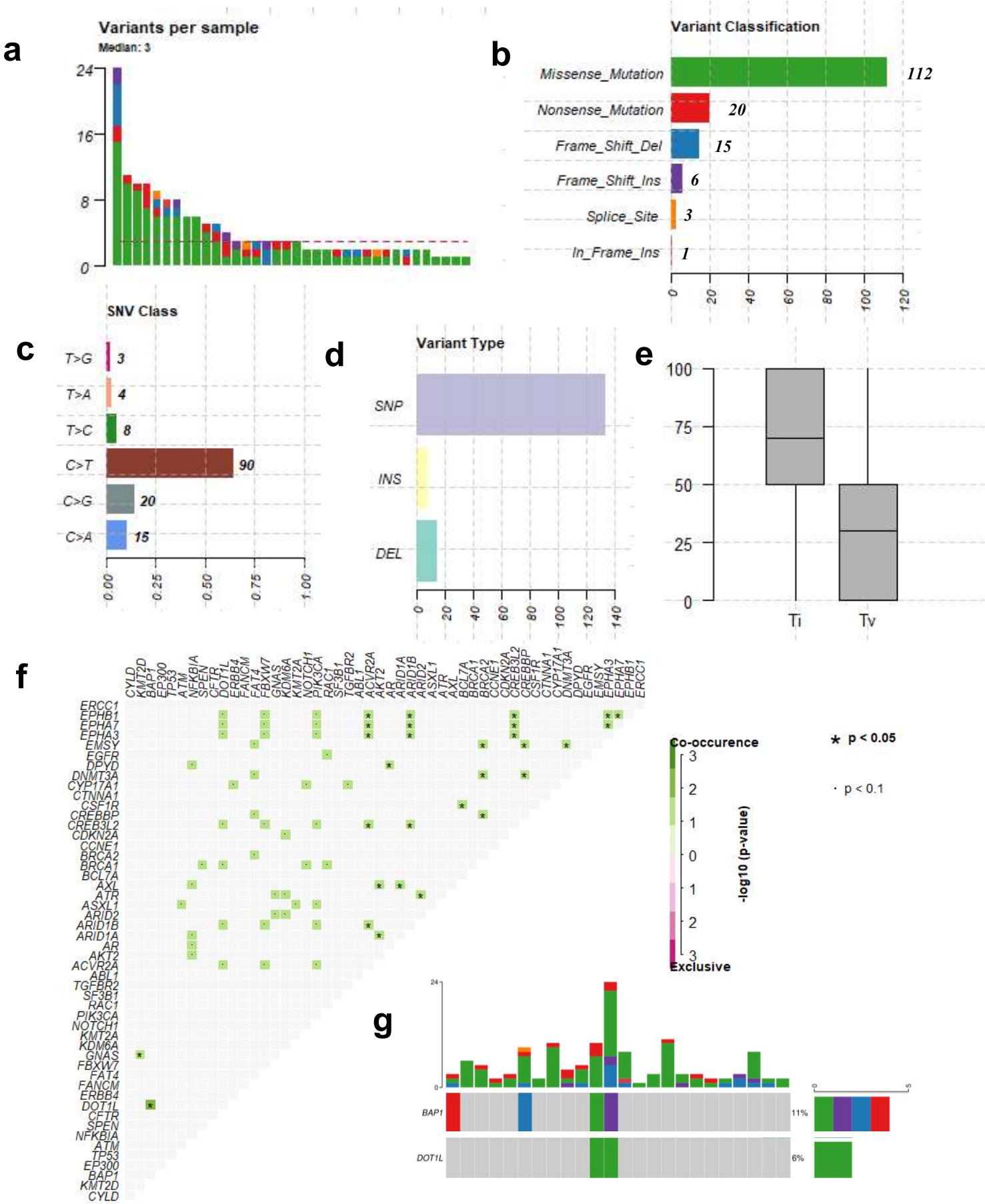

Supplement: Supplementary file 2 — Additional file 2: Fig. S2. Mutational Landscape of NPC. (a-e) Mutational patterns and proportion of genetic alterations in nasopharyngeal carcinoma. (f) Statistically significant mutual exclusivity or co-occurrences among the identified genes using pairwise Fisher’s exact test. (g) Oncostrip plot showing the detailed co-occurrences of BAP1 and DOT1 L in NPC. [file 10020_2022_479_MOESM2_ESM.pdf]
